# Supporting medical students to support peers: a qualitative interview study

**DOI:** 10.1186/s12909-022-03368-w

**Published:** 2022-04-20

**Authors:** Jane Graves, Eleanor Flynn, Robyn Woodward-Kron, Wendy C. Y. Hu

**Affiliations:** 1 School of Medicine, Western Sydney University, Sydney, Australia; 2 Melbourne Medical School, University of Melbourne, Melbourne, Australia

**Keywords:** Peer support, Student support, Medical students, Mental health, Wellbeing

## Abstract

**Background:**

Students may be the first to recognise and respond to psychological distress in other students. Peer support could overcome medical student reluctance to seek help despite their high rates of mental ill-health. Yet, despite the adoption of peer support programs, there is little evidence of impact on students. Peer support programs may assume that medical students accept and view peer support positively. We explored these assumptions by asking students about their experiences and views on peer support.

**Methods:**

Qualitative semi-structured interviews exploring peer support experiences and views on peer support were conducted with ten medical students at two contrasting medical schools. Informed by a constructivist stance, interview transcripts underwent thematic analysis.

**Results:**

Three groups of themes were identified: participants’ experiences of peer support encounters, concerns about providing support, and views on students’ roles in peer support. Participants readily recalled signs of peer distress. Encounters were ad hoc, informal, and occurred within relationships based on friendship or by being co-located in the same classes or placements. Concerns about initiating and offering support included lack of expertise, maintaining confidentiality, stigma from a mental health diagnosis, and unclear role boundaries, with implications for acceptance of student roles in peer support.

**Conclusions:**

Our study emphasised the centrality of social relationships in enabling or discouraging peer support. Relationships developed during medical studies may anticipate the collegial relationships between medical professionals. Nevertheless, only some students are willing to undertake peer support roles. We suggest different strategies for promoting informal peer support that can be offered by any student, to those promoting formal support roles for selected students. Future research focusing on the impact for both the students who receive, and on the students who provide peer support is called for.

**Supplementary Information:**

The online version contains supplementary material available at 10.1186/s12909-022-03368-w.

## Background

The prevalence of depression or depressive symptoms in medical students is estimated to be 27%, and suicidal ideation 11%, higher than in the general population [1, 2]. Mental illness stigma continues to be a major barrier to seeking support and treatment; only 20% of medical students state they are comfortable to seek help for depression or anxiety [3]. Embarrassment, and concerns regarding lack of confidentiality or privacy, the competitive nature of medical education and career progression, and the perfectionist qualities of many medical students contribute to a persistent reluctance to seek help [4–6].

Such barriers could be transcended by peers supporting each other. Studies show repeatedly that friends and peers tend to be the first source from whom students seek support, and peers may be the first to detect early signs of distress [7–9]. Peer support encompasses a range of interpersonal helping behaviours undertaken by non-professionals to help others of the same status [10]. Such roles and behaviours are consistent with competencies in self-care and care for others, encoded in international standards for medical graduate outcomes and in professional codes of conduct [11, 12].

It is argued that student involvement in peer support raises awareness of mental health problems and reduces mental illness stigma [13, 14]. Medical student societies have developed mental health campaigns to improve well-being and empower students to look out for their peers [15]. Peer support could facilitate early help-seeking and be an entry point for further professional advice [16].

Accordingly, universities have adopted formal peer support programs [17] to train students in mental well-being support [18]. Such programs may include structured skills based activities in mental health education, facilitation of topic-based discussions, mentoring, exercise and meditation [19]. Proponents argue that such programs positively impact on medical students [14, 20–22]. Formal peer support can also complement informal peer support, or student self-initiated helping behaviours in response to being aware of a peer in distress. Whilst a peer support role can be perceived as meaningful, previous research shows both mixed and promising results in terms of beneficial outcomes for the provider and receiver of support [23]. Recent systematic reviews have not found any robust evidence that peer support improves mental wellbeing among university students. In both reviews, few studies were of adequate quality to meet inclusion criteria [18, 24]. The authors concluded that rigorous evaluation to identify evidence for peer support programs was needed [18].

High quality evidence has been hampered by programs tending to be small scale, short term and reliant on feedback from voluntary participants as outcome measures [14, 20, 21]. One reason for lack of sustainability may be program dependence on individuals with a personal interest in mental health, while engagement and credibility with the broader medical student cohort may be limited. Together with the lack of evidence of effect from peer support, there appears little evidence about how the general student cohort, rather than student volunteers, regard peer support and peer support programs, and about how support is enacted informally.

We therefore sought to build this evidence by asking students:

- How do medical students identify and support peers in distress?
- What are students’ views on peer support and on students’ roles in supporting peers?

## Methods

We conducted a qualitative semi-structured interview study, and report our findings according to accepted reporting guidelines for qualitative research [25]. For the study, we adopted a constructivist approach that seeks to understand the participants’ experiences from their perspective, and their interpretations of reality. Our participants were medical students enrolled at two contrasting Australian medical schools located in urban settings in different states; one offered a graduate entry four-year, and the other an undergraduate entry five-year program. Both medical schools had active medical student societies with student mental wellbeing interest groups. Wellbeing learning activities were part of the regular teaching schedule, but there was no formal peer support program for medical students in either school.

### Data collection and analysis

Students were purposively sampled from the clinical years (3^rd^ to final year) of the general cohort as well as from mental wellbeing interest groups. These more advanced students and students with an interest in mental wellbeing were invited due to topic sensitivity and their greater likelihood of having encountered situations where support was, or could have been, provided. We conducted 10 in-depth interviews, a purposivelyselected sample, varying in age, years of study, interest in student mental wellbeing, and in healthcare and educational systems, from a potential study population of approximately 900 students in the clinical years of both medical schools. The mean age was 24.2 with a standard deviation [SD] of 4.3. To prevent inadvertent identification, recruitment and interviews were conducted by researchers in the other medical school (JG, EF) and data de-identified by the interviewer before analysis. Ten audio-recorded telephone interviews of up to 40 min were conducted and transcribed verbatim. As peer relationships and trust in wellbeing initiatives impact medical student wellbeing, [26] our interview questions focused on experiences with peers who had struggled, opportunities and issues with providing peer support and peer support roles. The interview schedule was trialed and refined after piloting (see Additional File 1: Interview Questions).

All researchers were experienced in providing academic and pastoral support to medical students, and with teaching mental wellbeing in their medical programs. EF, RWK and WH had conducted staff and student workshops, and qualitative research on student support and mental wellbeing. EF and WH were medical doctors, JG a chiropractor and RWK a linguist. All brought their professional and disciplinary backgrounds to the data analysis and interpretation, and support evidence-based interventions for student well-being.

Thematic analysis [27] was conducted, assisted by NVivo 12 ©. Data underwent preliminary analysis by two researchers (JG, EF) and initial codes examined and refined with all researchers before further coding and confirmation of themes through an iterative process of identifying exemplar extracts, drafting and re-drafting of theme descriptors. Data collection continued until no new themes emerged.

The study was approved by the Research Ethics Committees of the University of Melbourne (ID 1,238,315.2) and Western Sydney University (ID H9989) and all methods were carried out in accordance with relevant guidelines and regulations**.** Informed consent for study participation was obtained from all subjects who were all over 18 years of age.

## Results

For privacy, only aggregate participant characteristics are presented, and pseudonyms reflecting the background of the school cohorts used throughout. Ten interviews were conducted; six of the ten students identified as female (Anika, Emily, Mariam, Nadine, Shakti, Tho) and four as male (Abdul, Colin, Nikhil, Sasha). Three groups of themes were found: Peer Support Experiences, Initiating and Providing Support, and Peer Support Roles. These are described in turn with illustrative quotes.

### Peer support experiences

Participants described situations and encounters where there was an opportunity for peer support to be offered or was offered. Peer support was nearly always mediated by existing social interactions, whether through shared learning experiences or friendship.

#### Awareness of stress and distress

Participants described being increasingly aware of psychological stress and distress in their peers, noting signs such as being absent, appearing withdrawn or distressed and admitting to negative thoughts:

…he was not going to uni as much…he was sleeping more…he just seemed a bit - maybe his mood or the way he carried himself - seemed a bit down. Colin

Students did not view teaching or professional staff as sources of advice or assistance for mental wellbeing concerns; some expressed astonishment at this idea:

Even though the admin staff at the hospital are super lovely, I can’t imagine ever going to talk to them about a personal issue. Anika

Peers, in contrast, could be well-placed to identify and support other students in need, especially through existing social networks:

… it’s even better combated when students are on board as well, because we have direct connections to everyone. Colin

When peer support was offered, it was within the context of relationships based on spending time together in the course, or on existing friendship groups. Initiating contact through social media was described, but most encounters were in person, often through casual conversations:

We would get the train home together at the end of the day and she was often in tears so in the end I sent her a Facebook message that said, ‘Hey, if you ever want to talk about it, I’m here and I hope you’re doing okay, and if you want to hang out this weekend,’ and just that kind of casual friendly message. Yeah, and she just wrote back saying, ‘Thanks, that’s so nice’ Tho

…mostly just like commiserating…being like – brush it off and like, you know, tomorrow’s a new day and you don’t know, it might be way better tomorrow. Nikhil

These contacts signaled care and concern, and assisted decision making by exploring options. Occasionally students described more practical advice:

…we had a long chat as well, and we devised sort of like an action plan of how we would get his work done and also like what sort of help he should seek externally, to make sure he was like safe and in a good headspace. Abdul

#### Evolving friendships

Friendships developed easily within the small group learning environment of the early on-campus years. The value and meaning of these new relationships to medical studies and to becoming a doctor were not realised until later:

I went into medical school thinking I didn’t need to make any friends because I already had friends….but I made amazing friends for life, and I think that’s been the most important and the most beneficial thing for me, getting through this degree. Anika

Connections within friendship groups changed with course progression, with on-campus friendships becoming more distanced as students entered the clinical years and experienced the relative dislocation of clinical placements:

….it’s a bit isolating, I think, being put in – you know, one clinic for one day and then another clinic for another day. Sasha

Long hours in clinical settings, together with reduced opportunity for extracurricular social connections led to a new camaraderie, modelling the support medical professionals receive from colleagues, united by shared experiences and challenges.

But someone used the term, ‘prison friends’ as in you spend so much time with everyone here. Emily

However, not all students could easily access informal support from friends and peers. Three participants voiced concerns about socially isolated students:

I think the best thing is just to have a good group of friends around you, which I'm personally lucky to have, but not everyone does have that, which is a shame. Shakthi

### Initiating and providing peer support

Overall, more concerns were raised than expressions of interest in supporting peers. These concerns were interrelated, and interacted to express a general reluctance to offer peer support. They included: feeling ill-equipped to help, confidentiality and privacy, mental illness stigma and uncertain boundaries.

#### Lack of expertise

Participants felt for the distressed student and that they should help, but also that they lacked expertise because circumstances were beyond their level of comfort or expertise.

Like someone has told me that they’ve had suicidal intentions, and I thought that I was just not equipped…Nadine

…like I wanted to help but there was nothing I could do. It was beyond my expertise. Shakthi

#### Safeguarding confidentiality and privacy

Yet, despite recognising that more help was needed, participants repeatedly emphasised the importance of maintaining peer confidentiality.

That seems like the rules of a good friend is someone tells you something in confidence, they wouldn’t tell someone else. Anika

While a medical emergency justified breaching confidentiality, the choice of action was moderated by privacy concerns:

I’d probably try and call someone, like the mental health service kind of thing, rather than go to a hospital. Because I don’t know how I would feel if I was really struggling and then someone took me to the hospital that I was going to work at.” Sasha

#### Mental illness stigma

Pervasive stigma amongst peers about mental illness, and the desire to maintain face increased the need for confidentiality, driven by concerns about impact on future careers. Paradoxically, mental health education – especially when framed as disease – augmented this stigma, rather than legitimizing the symptoms:

…once we learn about mental illness, then, I think it would be a huge step for any one of my friends to say that they actually have one of those, like an actual diagnosis. Emily

Students described expectations to maintain face through a calm professional persona, knowing that public demonstrations to the contrary would be long remembered:

hopefully, we’re moving towards not having so much stigma around mental health but if someone’s only real recollection of you is you like sobbing uncontrollably in the emergency room…Sasha

However, peers could also reduce stigma, by normalizing the experience of stress and distress through self-disclosure:

…just knowing that someone else is struggling with the same thing, probably makes you feel better and it makes me feel better…people look around and they think that everyone else has just got it together and they don’t really. Sasha

#### Uncertain boundaries

To participants, the boundaries between offering and not offering peer support were indistinct. For some, reluctance to be involved in peer support was based on an assumption that counselling or knowledgeable advice was required. Others were able to describe limits, expressing more confidence in offering support:

…we don’t feel like we’re professionals and, maybe, our role or someone might see their role as general support and general friendliness. But if things are serious, then, continue to do that, but you need to escalate it to someone else. Emily

Another boundary was created by the absence of friendship or shared learning experiences on which to initiate contact. Most were yet to adopt professional behavioural norms about care for colleagues, so this boundary could become an additional barrier, especially if the struggling student was not attending classes or was withdrawn:

... some people don’t make friends as easily as others and then those people are isolated, and they don’t get the support that they need. Sasha

### Peer support roles

While students valued peer support in the context of friendship groups, they raised concerns about formalised programs. These included the selection of “peer support officers”, perceptions of lack of authority, and the potential for breaches of confidentiality and of negative emotional impact on students in these roles.

I think it should be an informal role, I think it has to do with sort of, friendship and supporting your friends. Sasha

Despite the participants’ overall reluctance to be involved in peer support, there were some willing individuals, who suggested various curricular additions for promoting peer support (see Additional File 2: Curricular strategies suggested by participants to facilitate peer support).

## Discussion

Our study adds to previous research on barriers to seeking help [5, 28] by finding that these barriers also exist for providing help, but can be mediated by social relationships. During medical school, early friendship based relationships appear to evolve into emergent professional relationships. The necessity for existing relationships or professional norms before peer support can occur may help explain the lack of intended effect and limited longevity of peer support programs. Our findings suggest that differentiated educational interventions for the general student cohort, and for selected students to become peer support “mentors” are appropriate. These findings are explored below.

### Centrality of trusting relationships

Our study extends previous findings that peers are likely to be the first to notice and respond to distressed peers by highlighting how these expressions of concern germinate and grow from within social relationships. These relationships often begin with new friendships formed in the early years of the course, and evolve with shared learning experiences during the relative isolation of placements in the clinical years. These later relationships appeared prototypical of workplace based relationships between medical professionals and signal an emergent professionalism. Collegial relationships are an important vehicle for peer support in the medical profession, may protect against burnout, and help manage distress from medical mishaps and adverse outcomes [29, 30]. Our study suggests that placement timetabling and learning activities could be purposively designed to promote collegial relationships, rather than being incidental enablers experienced as virtual incarceration or “prison friends(hip)”. For example students could be placed together with enough time to allow development of collegial relationships, facilitated by discussion on the value and meaning of working together as peers on learning challenges in hierarchical workplaces [31]. By emphasizing the social and reciprocal nature of collegiality, students can be reminded of how such behaviours enact and preserve professional norms and thus the integrity of the profession [32]. This may help counter pervasive student fears about risk to career from disclosing mental health concerns, which can be reinforced by over-emphasis and limited knowledge about legal obligations to protect the public by reporting impaired colleagues [33].

Relationships appeared critical to initiating peer support, suggesting why only a few participants supported students being appointed to peer support roles, and then with caveats. Even with careful selection by the medical school, perceived lack of authority, credibility and risk of unwanted disclosure could compromise the role. In short, without a foundation of trust, students were unlikely to entrust peers with their fears and mental health concerns. Likewise, our earlier research suggests that students are unlikely to engage in school support programs when they mistrust the school to act in their interests.

### Lack of intended effect and longevity

Absence of trusting relationships also suggests why formal peer support programs lack longevity [18]. Their implementation in private industry and government workplaces with outcome measures such as reduced sick leave after workplace trauma [34] suggests they are designed for organisational performance, rather than for the wellbeing of individuals, or for a higher purpose such as service to the profession.

In medical schools, peer support programs may be delivered by members of student interest groups [14, 35] . This is consistent with our finding that not all students are willing or even appear interested in being involved in supporting peers. When committed individuals graduate or become focused on study or other responsibilities, such programs may become unsustainable. Succession planning of programs which have been evaluated to be effective should be included in their design.

Another unintended consequence is the potential for student support providers to bear additional emotional burdens, intensifying stress from balancing service with study and their own mental health concerns. While participating in peer support may promote resilience in the students who provide support [36], participants drew attention to the need for on-call and regular advice and training for students who undertake these formal roles.

We found an unintended consequence of mental health curricular content was further reluctance to attach a diagnosis to mental health symptoms, sometimes expressed in the interviews as a hesitation to use or avoid medical terminology when describing peers. Rather than legitimising symptoms, such labels denoted a stigmatising illness. This reluctance could be further explained as projections of the participants’ own mental health concerns, with the confidentiality of a research interview being insufficient for openness. Self-disclosure of mental ill-health and its impact by peers, near-peers and role models may reduce stigma [37].

Our findings suggest informal peer support can be valuable but different strategies are needed for the general student cohort, so they are informed and prepared for informal encounters, compared to selected students who are better suited to formal and more proactive peer support roles. Socially isolated students who likely need more proactive support and who may not come to the attention of staff, could be an important focus for students in peer support roles (see Table 1). Strategies need to be tailored to context; including student cohort characteristics, presence of academic and professional staff sponsors, availability of mental health services both within and outside the medical school, university and health service policies and procedures.

**Table 1:** Education and training for promoting informal and formal peer support

**For all students, to promote informal peer support encounters**
• Information about mental health symptoms, guidance on when, how and who to refer to, specific to context
• Scripts and practice in expressing concern and broaching mental health topics
• What to do in emergencies, red flags
• Self-disclosure by peers, near-peers and role models emphasizing strengths-based management and careers advice
• Accurate information about professional obligations and codes of conduct
• Exploration of the value of peer relationships as emergent professional collegiality
**For selected students in formal peer support programs**
In addition to above
• Explicit selection criteria e.g. previous experience and training, accessibility and ability to contribute, absence of vulnerability, academic performance not at risk
• Role boundaries; when support should be paused, when to seek case advice, refer, how and to whom, specific to context
• Enhanced training in mental health responses and reaching socially isolated students
• Academic and/or clinical advice on demand 24/7
• Program design includes succession planning

### Limitations

Although our sample could be interpreted as small in number compared to the study population, participants were a purposively selected for maximum variation. Despite differences in their support of peer involvement, participants raised common concerns about peer support. Our findings relate specifically to the two medical school contexts in which the data was collected, thus readers should interpret these findings through the lens of their specific teaching and learning environment.

The researchers were teaching academics – although not involved in assessment—and known to work in student support, so may have elicited socially desirable responses. However, even interested students expressed qualms about peer support and our findings differed from previous studies conducted in the same medical schools with academic and professional staff participants. Professional [38] and academic [39] staff reported frequently providing pastoral and learning advice to students and to being the first to be contacted by students with concerns. In the current study students were surprised that staff could be considered a primary source of support. Together with Byrnes’ wellbeing study with student participants [26], these differences underpin the importance of gathering all perspectives when investigating complex or sensitive social interactions. This context dependent complexity suggests a comprehensive case study approach, or a design based interventional study [40] analysing data from a variety of sources could best uncover mechanisms of student wellbeing support in each context and address the lack of practical evidence for effective peer support.

## Conclusion

Our study suggests that initiating and providing peer support does not come easily to many medical students unless there are pre-existing social relationships. Informal peer support encounters may herald the emergence of medical professional behavioural norms and could be sought as teaching moments in professionalism. We suggest differentiated educational strategies, tailored to all students on how to support peers, and to selected students who wish to undertake more formal roles. Our findings support evidence-based program design, and any interventions must be rigorously evaluated for intended and unintended impacts on the students who receive, and who provide, support to their colleagues. We propose future research to explore the finding that informal peer support between medical students is dependent on friendship, which may limit its use. Additionally, we propose further studies to evaluate the proposed peer support training, including informal peer support training for all medical students coupled with formal peer support training for interested students.”

## Supplementary Information

**Additional file 1.** Interview questions.

**Additional file 2. **Curricular strategies suggested by participants to facilitate peer support.

## Data Availability

The datasets created for this study are available from the corresponding author on reasonable request.

## References

[1] Moir F, Yielder J, Sanson J, Chen Y (2018). Depression in medical students: current insights. Adv Med Educ Pract.

[2] Rotenstein LS, Ramos MA, Torre M, Segal JB, Peluso MJ, Guille C, Sen S, Mata DA (2016). Prevalence of depression, depressive symptoms, and suicidal ideation among medical students: a systematic review and meta-analysis. JAMA.

[3] BeyondBlue (2013). National mental health survey of doctors and medical students.

[4] AMA (2020). Health and wellbeing of doctors and medical students 2020.

[5] Chew-Graham CA, Rogers A, Yassin N. ‘I wouldn’t want it on my CV or their records’: medical students’ experiences of help-seeking for mental health problems. Med Educ. 2003;37(10):873–80. 10.1046/j.1365-2923.2003.01627.x 12974841

[6] Sayburn A. Why medical students' mental health is a taboo subject. BMJ. 2015;350:h722.

[7] Cyr C, McKee H, O’Hagan M, Priest R (2016). Making the case for peer support: report to the peer support project committee of the mental health commission of Canada.

[8] Thornicroft G, Mehta N, Clement S, Evans-Lacko S, Doherty M, Rose D, Koschorke M, Shidhaye R, ReillyApos C (2016). Evidence for effective interventions to reduce mental-health-related stigma and discrimination. Lancet.

[9] Thompson D, Goebert D, Takeshita J (2010). A program for reducing depressive symptoms and suicidal ideation in medical students. Acad Med.

[10] Moir F, Henning M, Hassed C, Moyes SA, Elley CR (2016). A peer-support and mindfulness program to improve the mental health of medical students. Teach Learn Med.

[11] Australian Medical Council (2012). Standards for Assessment and Accreditation of Primary Medical Programs by the Australian Medical Council 2012.

[12] Frank J, Snell L, Sherbino J (2015). CanMEDS 2015 Physician Competency Framework.

[13] Baik C, Larcombe W, Brooker A (2019). How universities can enhance student mental wellbeing: the student perspective. High Educ Res Dev.

[14] Ahmed AK, Nault T, Rizos J, Taneja K, Kim GP (2020). Peer support: A medical student-driven mental health workshop. Med Educ.

[15] Dean JM, Cliff ERS, Freilich K, Gilfillan M (2016). A campaign to improve the mental health of medical students. Med J Aust.

[16] Johnston C (2015). Medical students talk openly about their mental health challenges. Can Med Assoc J.

[17] Kemp S, Hu W, Bishop J, Forrest K, Hudson JN, Wilson I, Teodorczuk A, Rogers GD, Roberts C, Wearn A (2019). Medical student wellbeing – a consensus statement from Australia and New Zealand. BMC Med Educ.

[18] John NM, Page O, Martin SC, Whittaker P. Impact of peer support on student mental wellbeing: a systematic review. MedEdPublish. 2017;7(3):170. 10.15694/mep.2018.0000170.1 PMC10701817 38074560

[19] Byrom N (2018). An evaluation of a peer support intervention for student mental health. J Ment Health.

[20] Redwood SK, Pollak MH (2007). Student-led stress management program for first-year medical students. Teach Learn Med.

[21] Lau KS, Siong KH, Tang HY, Cheng PW, Cheung KS, Chan SW, Lee PWH, Wong JGWS (2007). An innovative web-based peer support system for medical students in Hong Kong. Med Teach.

[22] Farber SB, Parlow SDG, Timmerman NP. A Peer-Based Approach to Reducing Stigma and Improving Mental Health Support for Medical Students. University of Ottawa journal of medicine. 2017;UOJM ePub:1-2.

[23] Tong A, Sainsbury P, Craig J. Consolidated criteria for reporting qualitative research (COREQ): a 32-item checklist for interviews and focus groups. Int J Qual Health Care. 2007;19(6):349–57. 10.1093/intqhc/mzm042 17872937

[24] Wall A, Lovheden T, Landgren K, Stjernswärd S. Experiences and challenges in the role as peer support workers in a Swedish Mental Health Context - an interview study. Issues Ment health Nurs. :1–12. ahead-of-print(ahead-of-print). 10.1080/01612840.2021.1978596 34569894

[25] Akinla O, Hagan P, Atiomo W (2018). A systematic review of the literature describing the outcomes of near-peer mentoring programs for first year medical students. BMC Med Educ.

[26] Byrnes C, Ganapathy VA, Lam M, Mogensen L, Hu W (2020). Medical student perceptions of curricular influences on their wellbeing: a qualitative study. BMC Med Educ.

[27] Braun Va. Successful qualitative research: a practical guide for beginners. In: Clarke Va, editor: London: London Sage Publications; 2013.

[28] Clement S, Schauman O, Graham T, Maggioni F, Evans-Lacko S, Bezborodovs N, Morgan C, Rüsch N, Brown JSL, Thornicroft G (2015). What is the impact of mental health-related stigma on help-seeking? A systematic review of quantitative and qualitative studies. Psychol Med.

[29] Olson K, Marchalik D, Farley H, Dean SM, Lawrence EC, Hamidi MS, et al. Organizational strategies to reduce physician burnout and improve professional fulfillment. Current problems in pediatric and adolescent health care. 2019;49(12):100664. 10.1016/j.cppeds.2019.100664 31588019

[30] Bjørke-Bertheussen J Fau - Hagerman A, Hagerman A Fau - Hegelstad WTV, Hegelstad Wtv Fau - Fredriksen KJ, Fredriksen KJ: Collegial support when patients take their own life. LID - FAU - Bjørke-Bertheussen, Jeanette. (0807–7096 (Electronic)). 10.4045/tidsskr.17.0908

[31] Chou CL, Teherani A (2017). A foundation for vital academic and social support in clerkships: learning through peer continuity. Acad Med.

[32] Cruess SR, Johnston S, Cruess RL. “Profession”: A Working Definition for Medical Educators. Teach Learn Med. 2004;16(1):74–6. 10.1207/s15328015tlm1601_15 14987179

[33] Medical Board of Australia (2020). Good medical practice: a code of conduct for doctors in Australia.

[34] Peer support reduces sick leave after safety incidents https://www.ohsalert.com.au/nl06_news_selected.php?act=2&nav=11&selkey=53390&utm_source=daily+email&utm_medium=email&utm_campaign=subscriber+email&utm_content=article+headline&utm_term=Peer%20support%20reduces%20sick%20leave%20after%20safety%20incidents

[35] Changing Medical Student Wellbeing – A peer support initiative https://www.umdoseofreality.org/7792/changing-medical-student-wellbeing-a-peer-support-initiative

[36] Identifying good practice among medical schools in the support of students with mental health concerns. http://www.gmc-uk.org/Identifying_good_practice_among_medcal_schools_in_the_support_of_students_with_mental_health_concerns.pdf_52884825.pdf

[37] Martin A, Chilton J, Gothelf D, Amsalem D (2020). Physician self-disclosure of lived experience improves mental health attitudes among medical students: a randomized study. J Med Educ Curric Dev.

[38] Flynn E, Woodward-Kron R, Hu W (2016). Training for staff who support students. Clinical Teacher.

[39] Hu WCY, Woodward-Kron R, Flynn E (2019). Educator as diagnostician, judge and confidant: a positioning analysis of medical student support encounters. Adv Health Sci Educ.

[40] Dolmans DHJM, Tigelaar D. Building bridges between theory and practice in medical education using a design-based research approach: AMEE Guide No. 60. Medical teacher. 2012;34(1):1-10. 10.3109/0142159X.2011.595437 22250671

