# Supplementary material for: Supporting medical students to support peers: a qualitative interview study
Source: BMC Med Educ. 2022 Apr 20;22:300. doi: 10.1186/s12909-022-03368-w (PMC9027875; doi:10.1186/s12909-022-03368-w)
Supplement: Supplementary file 1 — Additional file 1. Interview questions. [file 12909_2022_3368_MOESM1_ESM.docx]

**Additional file 1: Interview questions**

**Background**

1. Can you please tell me what year you are in?
   1. What did you do prior to entering medicine?
   2. Have you had any experience with supporting peers prior to entering medicine?
   3. Have you received any training about peer support? If so, what?
   4. In what ways do medical students become aware of peers who are struggling?
   5. What concerns cause students to struggle?

**Experiences of peer support**

1. Have you had any direct experiences with peers who have struggled during their medical studies?
   1. If so, can you tell me about one of your experiences, from the time that you found out that the peer was struggling, and what happened? How did it turn out?
   2. What was your role in this situation?
   3. Looking back on it now, would you have dealt with this differently? What would have helped you to better deal with this situation?

**Student roles in peer support**

1. What do you think is the role of students in peer support?
   1. Should it be a formal or informal role?

**Closing**

4 Are there any other points you would like to make about improving how students are supported during their training and education?
