# Supplementary material for: Supporting medical students to support peers: a qualitative interview study
Source: BMC Med Educ. 2022 Apr 20;22:300. doi: 10.1186/s12909-022-03368-w (PMC9027875; doi:10.1186/s12909-022-03368-w)
Supplement: Supplementary file 2 — Additional file 2. Curricular strategies suggested by participants to facilitate peer support. [file 12909_2022_3368_MOESM2_ESM.docx]

**Additional file 2: Curricular strategies suggested by participants to facilitate peer support**

| **Strategy** | **Example of strategy** | **Participant*** | **Quote** |
| --- | --- | --- | --- |
| Training | Mental Health,  Suicide Prevention training.  Peer support workshops.  Online mental health training modules.  Tutorials which include role-play and information about the university support services, mental health red flag education and when to escalate for support. | Tho  Mariam  Tho  Sasha  Nadine | That was a two-day workshop for Mental Health First Aid**, which I found really, really helpful actually  Definitely training in how to approach a student who is in trouble and you know, the steps to take with them, so allowing them to seek help, would be beneficial.  The discussion at the (peer support) workshop with [Professor] and [Psychiatrist] was actually quite good  An online mental health training module…was actually quite good  …like regular workshops on how to handle situations where there’s a bit more roleplaying involved. Like where you have to break it to a friend that you may need to tell staff, or you recommend that they tell staff |
| Accessing available support services | Factsheet, booklets and online flowsheets, information about staff availability.  Wellbeing staff member independent from teaching and assessment.  Student-led wellbeing group. | Nadine  Nikhil  Anika  Mariam  Tho | …….there’s like more adequate flow sheets of like support pathways  So it’s worth some training in regards to what kind of services we can actually access as students. So we can help each other.  As a student, you sort of get shuffled around from the university to the hospital and there are tonnes of staff employed by the hospital, employed by the medical school, and it’s always quite vague and confusing to who the people are  Say if they have a friend who’s quite – has a bit of suicidal ideation, often students don’t want to go and speak to an academic about that. is always – helps students feel a bit more secure, in seeking help  And the Wellbeing Committee I think will definitely help. |
| Address barriers to help seeking | Stigma  Confidentiality and mandatory notification | Tho  Mariam | ….like the main issue is really stigma, like in terms of mandatory reporting as well as general sort of stigma.  So perhaps more information about mandatory reporting and how confidentiality is kept? Like very prominently on the website if there’s a website that’s promoting like – it’s offering services, and having it very, very clear that these services remain anonymous, that sort of thing, because otherwise people just won’t use them |
| Buddy system | Peer support program | Nikhil  Nikhil | …… a student mentoring system. Kind of like a buddy system, like you may have had in primary school, where, you know, every first year will be assigned to a second year buddy, and every second year has a third year buddy.  …..developing a kind of contract, or agreement, for this buddy system. To outline kind of – boundaries of confidentiality |

* Pseudonyms are used throughout

**Mental Health First Aid (MHFA) teaches participants how to “assist people who are developing a mental health problem, experiencing a worsening of an existing mental health problem or in a mental health crisis, until appropriate professional help is received or the crisis resolves”

(Mental Health First Aid 2019) Take a Course. Mental First Aid Australia. Retrieved from http:mhfa.com.au May, 2021
